# Interorganellar Ca^2+^ Flux Assessment by Flow Cytometry Reveals an Altered Mitochondrial Ca^2+^ Homeostasis in Circulating Lymphocytes of STEMI Patients

**DOI:** 10.1016/j.jacbts.2025.101430

**Published:** 2025-12-22

**Authors:** Camille Brun, Juliette Geoffray, Florentin Moulin, Sylvie Ducreux, Gabriel Bidaux, Thomas Bochaton, Melanie Paillard, Claire CROLA DA SILVA

**Affiliations:** a University Claude Bernard Lyon1, CarMeN Laboratory-IRIS Team, INSERM, INRAE, Bron, France; b Cardiac Intensive Care Unit, Hospices Civils de Lyon, Bron, France

According to the World Health Organization, 17.9 million people in the world develop cardiovascular diseases each year. Despite the increasing effectiveness of patient care, the morbidity/mortality of ST-segment elevation myocardial infarction (STEMI) patients remains high, with a strong evolution toward heart failure (HF). Because cardiac biopsy obtention is not possible in STEMI patients, inflammation is mainly studied at the systemic level, notably by assessing circulating sera biomarkers.^1^ Peripheral blood mononuclear cells (PBMCs) have recently emerged as complementary biomarkers, because they display a molecular and functional signature of the tissue alteration in some cardiovascular diseases, notably HF.^2^ Maintaining Ca^2+^ homeostasis in PBMCs is crucial to their immune cell function.^3^ Few studies used flow cytometry to study Ca^2+^ fluxes in PBMCs and mainly focused on the cytosolic compartment.^4^ Thus, we aimed to develop a multiparametric flow cytometry protocol to study interorganellar Ca^2+^ fluxes in PBMCs from control donors and STEMI patients.

**Figure 1:**
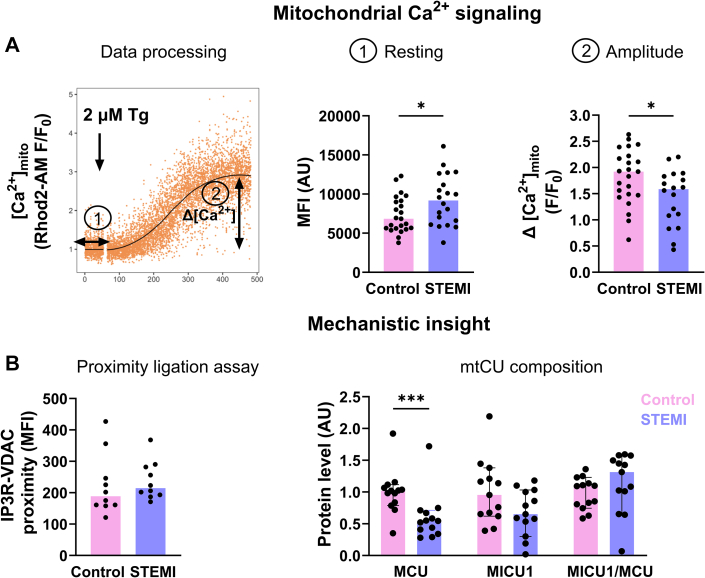
Mitochondrial Ca^2+^ Signaling in Lymphocytes (A) Flow cytometry data analysis of mitochondrial Ca^2+^ fluxes in human lymphocytes using R. Resting: median fluorescence intensity (MFI). F/F_0_ ratio was calculated to obtain a normalized curve. Thapsigargin-induced Ca^2+^ amplitude: difference between maximal peak and resting level (n= 18-23/group). (B) Quantification of IP3R (ab5804)-voltage-dependent anion channel (VDAC1) (ab14734) proximity in lymphocytes (n = 12 /group). Immunoblotting quantification in peripheral blood mononuclear cells of MCU (HPA016480), its regulator MICU1 (HPA037479), and calculation of the MICU1/MCU ratio (n= 13/group). Bar graph displays the median value with each dot representing a subject. Mann-Whitney *U* test: ∗*P <* 0.05, ∗∗∗*P <* 0.001. mtCU = mitochondrial calcium uniporter; STEMI = ST-segment elevation myocardial infarction.

PBMCs were obtained from 23 control donors (French National Blood Service) and from 20 STEMI patients (collected in the first 24 hours after reperfusion at the Hospices Civils of Lyon, after informed consent and institutional approval-DC-2019-3464). After thawing and 1 hour of resting, 8 × 10^4^ cells were loaded with a mix of 1 μmol/L FuraRed-AM with 80 μmol/L sulfinpyrazone in complete RPMI medium for 30 minutes at 37 °C. After 2 washes, cells were then loaded with 5 μmol/L Rhod2-AM/0.02% pluronic acid, washed, suspended in complete RPMI medium to allow the de-esterification process for 30 minutes at 37 °C, and finally placed on ice before the flow cytometry recording. Interorganellar Ca^2+^ fluxes were studied pharmacologically in light scattering-based gated lymphocytes. After baseline registration for 50 seconds, the tube was quickly removed from the cytometer to add 2 μmol/L thapsigargin (Tg) as a single bolus with a vortex step before returning the tube to the flow cytometer (Figure 1A). R software (version 4.3.3) was used to develop a data processing program. Endoplasmic reticulum-mitochondria contact sites were quantified by Duolink flow cytometry proximity ligation assay, and the mitochondrial calcium uniporter protein composition by immunoblotting. Data are expressed as median with 25th-75th percentiles (Q1-Q3). The Mann-Whitney *U* test was used to compare donor and STEMI groups. Analyses were performed using GraphPad Prism version 10.2.0 (GraphPad Software), and a *P* value <0.05 was considered statistically significant.

Age (median 64.0 years [Q1-Q3: 52-75 years] vs 45.0 years [Q1-Q3: 36-49 years]; *P* < 0.001) and percentage of men (80.9% vs 45.8%; *P =* 0.029) were significantly higher in STEMI patients compared with control donors, respectively. As expected, C-reactive protein (median 2,249 ng/mL [Q1-Q3: 1,799-3,176 ng/mL] vs 391 ng/mL [Q1-Q3: 261-575 ng/mL]; *P* < 0.001) was significantly increased in STEMI patients compared with control subjects, respectively. In resting conditions, the median fluorescence intensity of Rhod2-AM was significantly increased in lymphocytes from STEMI patients compared with control lymphocytes (Figure 1A), suggesting an increase in the mitochondrial resting [Ca^2+^] level. To study the contribution of both SOCE and endoplasmic reticulum Ca^2+^ release, Tg was used in Ca^2+^-containing RPMI medium. Although cytosolic response did not differ between control and STEMI lymphocytes (median 1.0 AU [Q1-Q3: 0.7-1.2 AU] vs 0.8 AU [Q1-Q3: 0.6-1.2 AU]), respectively, a significantly reduced Tg-induced amplitude was reported in the mitochondrial compartment in lymphocytes from STEMI patients compared with control donors (Figure 1A). Without external Ca^2+^, to focus on the reticular Ca^2+^ stock, no significant difference was observed in the cytosol/mitochondria Tg-induced amplitude, suggesting a potential alteration in mitochondrial Ca^2+^ uptake signaling in STEMI lymphocytes. Proximity ligation assay targeting the reticular inositol triphosphate receptor and the mitochondrial porin voltage-dependent anion channel, both involved in reticulum-mitochondria Ca^2+^ coupling, did not show any difference (Figure 1B). However, immunoblotting of the mitochondrial calcium uniporter components, performed on PBMCs because of the small amount of biological materials, revealed a significant decrease in the pore-forming protein level, MCU, in STEMI PBMC, while the MICU1/MCU ratio remained unchanged (Figure 1B).

Here, we report the use of flow cytometry as a robust strategy to perform multiparametric analyses of Ca^2+^ fluxes in several compartments of human PBMCs. We demonstrated that lymphocytes from STEMI patients display a higher mitochondrial Ca^2+^ load and a reduced mitochondrial Ca^2+^ uptake under large cytosolic Ca^2+^ stimulation, suggesting an alteration of mitochondrial Ca^2+^ signaling. Although the endoplasmic reticulum-mitochondria Ca^2+^ coupling was unaltered in STEMI lymphocytes, a remodeling of the mitochondrial Ca^2+^ uniporter occurs in STEMI PBMCs, ie, a decrease in the pore-forming MCU protein level. A previous study has already documented a RyR Ca^2+^ leak in circulating lymphocytes and in cardiomyocytes in a mouse model of HF.^5^ In case of a shared molecular phenotype between PBMCs and the injured myocardium, PBMCs could thus represent an elegant alternative to study myocardial damage in STEMI patients. Our study presents limitations: the age and cardiovascular risk factor imbalance between the control and STEMI groups could account for the observed effect; and the lack of separation of individual leucocyte classes within the PBMCs precludes the attribution of changes to a specific subset. Larger studies are needed to determine the contribution of each lymphocyte subtype as a potential post-STEMI biomarker.
