# Supplementary figures and images for: Interorganellar Ca2+ Flux Assessment by Flow Cytometry Reveals an Altered Mitochondrial Ca2+ Homeostasis in Circulating Lymphocytes of STEMI Patients
Source: JACC Basic Transl Sci. 2025 Dec 22;10(12):101430. doi: 10.1016/j.jacbts.2025.101430 (PMC12861672; doi:10.1016/j.jacbts.2025.101430)

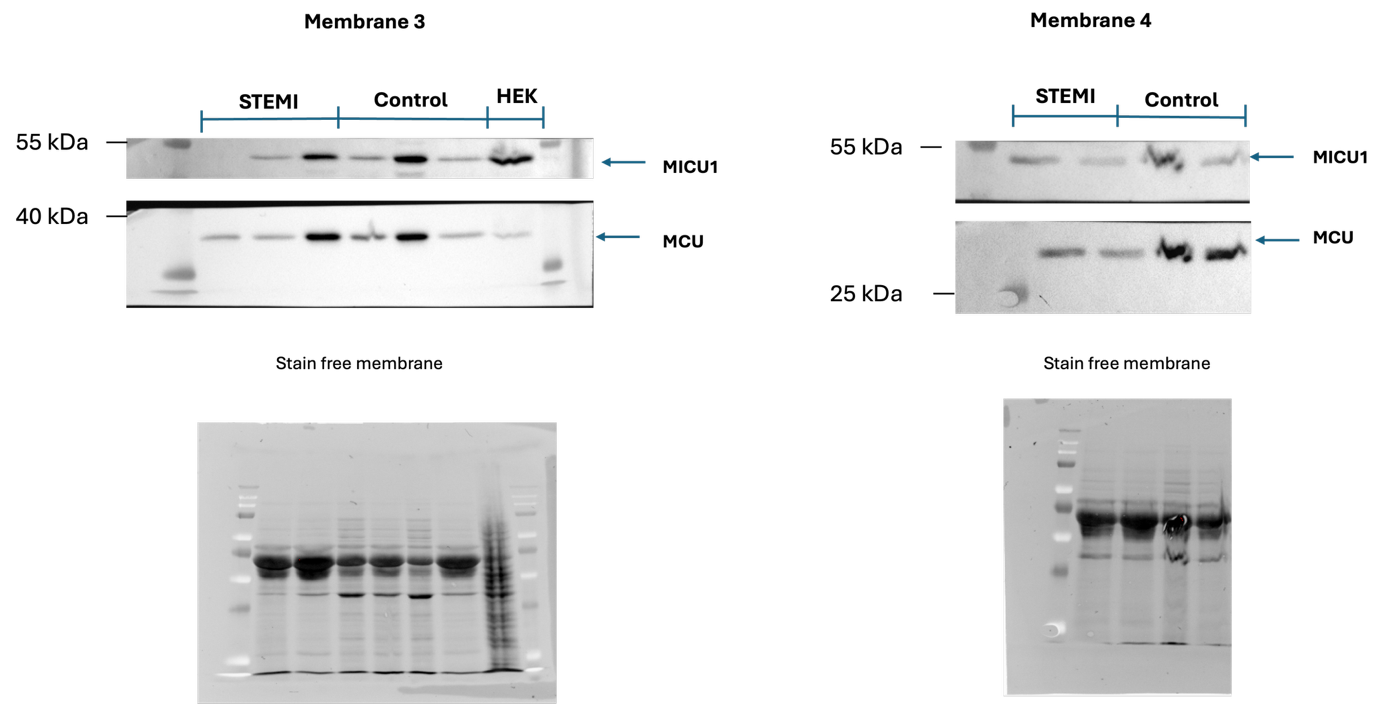

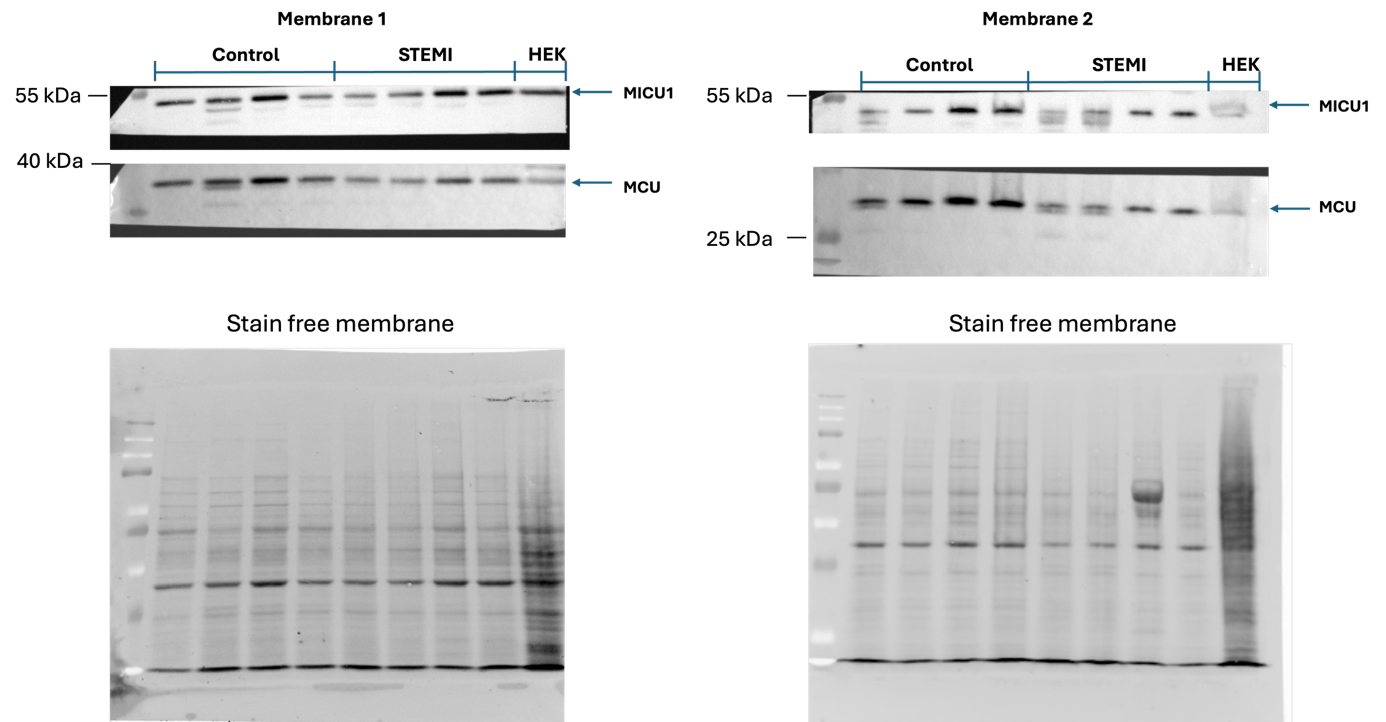

Supplement: Supplemental Figure 1 [file mmc1.docx]
